# Biocompatible Composite Materials Based on Porous Hydroxyapatite Ceramics and Copolymer of Lactide and Glycolide

**DOI:** 10.3390/ma14092168

**Published:** 2021-04-23

**Authors:** Daria Lytkina, Lothar Heinrich, Elena Churina, Irina Kurzina

**Affiliations:** 1 Department of Chemistry, National Research Tomsk State University, Lenin Ave. 36, 634050 Tomsk, Russia; darya-lytkina@yandex.ru (D.L.); lothar.heinrich@uni-muenster.de (L.H.); lena1236@yandex.ru (E.C.); 2 Institute of Biochemistry, University of Muenster, Wilhelm-Klemm-Str. 2, 48149 Muenster, Germany; 3 Department of Pathophysiology, Siberian State Medical University, Moskovsky tr. 2, 634055 Tomsk, Russia

**Keywords:** composite materials, hydroxyapatite, copolymer of lactide and glycolide, surface properties, in vitro, bone repair

## Abstract

The intensive development of reconstructive surgery and traumatology provides a stable demand for new materials for implants. Of particular interest are materials based on hydroxyapatite, which are chemically close to the elemental composition and structure of bone and have similar bioactive properties. The aim of this work was to obtain porous composite materials based on hydroxyapatite and a copolymer of lactide and glycolide with properties suitable for use as a material for bone implants. The phase and elemental composition of composites was investigated by infrared spectroscopy, X-ray diffraction, and X-ray photoelectronic spectroscopy methods, and it was established how the production process affects the composition of materials. Regularities of the formation of porosity by the methods of low-temperature adsorption of nitrogen, immersion in a liquid (determination of the pore space volume), measurement of the diffusion coefficient through the material (Franz cell), and surface properties of composite materials by the Hammett indicator method, by the lying drop method were revealed. Regularities were established between the surface properties and the composition of materials and their biocompatibility determined using monocytes isolated from human peripheral blood.

## 1. Introduction

The problem of reconstruction of bone defects resulting from trauma, surgical interventions and pathological changes in the body is relevant at the present time. Hydroxyapatite (HA) is one of the most suitable substances for its use as the main component of the material for the restoration of bone tissue, it is contained in the bone composition from 50 to 95% [1].

Ceramics are used to design biomaterials and are much less preferred than metals or polymers due to their fragility and low tensile strength. Nevertheless, bioceramics of phosphates are widely used as a biomaterial with high biocompatibility and integration into bone tissue, since it is the closest in composition to the mineral part of bones [2,3].

To improve the physicochemical and mechanical properties of hydroxyapatite, composites are obtained on its basis, in which polymers of various natures are used as a binder [4]. Of particular interest are copolymers of lactide and glycolide (PLGA), referring to a class of biodegradable polyesters, which are widely used in medicine [5]. Taking into account the existing disadvantages of certain groups of materials, low fracture toughness of ceramics and insufficient osteoconductivity of polymeric materials, we can conclude that the way out is to obtain composite materials that allow to level the disadvantages and enhance the advantages of individual components. Copolymer of lactide and glycolide may act as part of the materials as a component of replacing bone collagen. Composite materials based on HA and PLGA may be prepared by a variety of methods: solvent merging/particulate leaching method [6], foaming the suspension HA and PLGA [7], emulsification followed by freeze drying [8], electrospinning [9] and 3D printing [10]. Most of the materials based on polymer and HA are hydroxyapatite powder dispersed in the polymer [11], since such a material is resorbable, and a polymer matrix acts as a stiffener, which rather quickly loses its mechanical strength as a result of hydrolysis. To eliminate this feature, it seems possible to obtain a hydroxyapatite framework more stable to dissolution, followed by an improvement in its properties using a polymer. One of the key properties of a bone substitute is porosity, which promotes material ingrowth and vascularization [12] and a surface suitable for cell attachment and ingrowth. For this, a nontoxic pore former, sodium chloride, will be used, which will allow obtaining porosity sufficient for cell migration. The aim was to obtain biocompatible composite materials based on porous hydroxyapatite ceramics and a copolymer of lactide and glycolide and to establish what surface properties affect the biocompatibility of materials.

## 2. Materials and Methods

### 2.1. Synthesis of Hydroxyapatite

Synthesis of HA was carried out by the liquid-phase method under the influence of microwave radiation at pH ≈ 11 in accordance with the equation [13]

10Ca(NO_3_)_2_ + 6(NH_4_)_2_HPO_4_ + 8NH_4_OH → Ca_10_(PO_4_)_6_(OH)_2_ + 20NH_4_NO_3_ (1)

Freshly prepared solutions of calcium nitrate (C = 0.5 mol/L) and ammonium hydrogen phosphate (C = 0.3 mol/L) were mixed with stirring in equimolar volumes of 58.0 mL required to obtain the molar ratio of elements Ca/P = 5/3 characteristic of hydroxyapatite. By adding concentrated (25%, ⍴ = 0.9 g/mL) ammonia solution, the pH in the solution was adjusted in the range of 10–11. The reaction mixture was exposed to microwave radiation at a power of 110 W and a temperature of ~100 °C for 35–40 min. Then the resulting precipitate was defended in the mother liquor at room temperature for 48 h. The precipitate was filtered off and then dried in a drying oven (~20 h) at a temperature of 110 °C.

### 2.2. Obtaining Composite Materials GA-PLGA

The process of obtaining composite materials consisted of two stages: the first-obtaining a porous hydroxyapatite framework using a soluble or burnout additive; the second is the coating of the framework with a copolymer of lactide and glycolide to improve biocompatibility and mechanical properties.

The choice of pore-forming additives is due to their low toxicity and the possibility of their almost complete removal from ceramics; similar methods were also used in [14].

The calcination temperature was chosen to be 700 °C, which is 100 °C lower than the Tm of NaCl in order to prevent the NaCl from melting. Samples with a content of ˃50 wt.% NaCl also undergoes destruction upon ignition.

Hydroxyapatite and sodium chloride were sieved on a laboratory sieve with a mesh diameter of d = 0.001 cm, mixed until a homogeneous mixture was formed in the ratios of 90:10, 75:25, and 50:50. Next, by pressing (T = 25 °C, P = 200 atm) tablets (d = 19 mm, h = 1 mm) weighing 1 g were obtained. The obtained tablets were calcined in a muffle furnace at a temperature of 700 °C for three hours to obtain a compact frame material. Next, the tablets were soaked in distilled water for three days with water renewal every 12 h. The obtained porous ceramic framework made of hydroxyapatite was dried in air (12 h) and then in vacuum (12 h). As a result, ceramic frame materials were obtained with different porosity, which increases from HA (90) to HA (50).

To obtain a solution, PLGA was dissolved in chloroform with various concentrations (0.01–0.25 g/mL). Porous HA materials were immersed in a polymer solution and sonicated for 20 min to remove air bubbles from the substrate in order to obtain uniform coatings.

### 2.3. Physicochemical Research Methods

#### 2.3.1. Study of the Composition of Materials

The phase composition of the initial components and the obtained composite materials based on HA and PLGA was determined on an XRD-7000 diffractometer (Shimadzu, Kyoto, Japan), CuK_α_ radiation. Survey shooting was carried out in the range of reflection angles 2θ = 3–100 with a step of 0.05; accumulation time at point 3 s. The phases were decoded and identified using the ICDD diffraction database (PDF-2/Release 2012 RDB).

The spectra of the surface layer of samples with a thickness of about 2.5–15 microns of various materials were recorded on an FTIR-8300 FTIR spectrometer (Shimadzu) by attenuated total reflection technique. The spectra were recorded with a resolution of 4 cm^−1^ in the range 400–4000 cm^−1^. 

The chemical composition of the surface of the composite materials was studied by X-ray photoelectron spectroscopy (XPS) using the K-Alpha X-ray Photoelectron Spectrometer System (Thermo VG Scientific, Waltham, MA, USA). For the analysis, we used a monochrome X-ray source AlK_α_ with an X-ray spot 400 μm in size. The spectra were measured using an energy of 80 eV for the survey spectra and 30 eV for the ground level spectra. To determine the elemental composition of the surface, additional cleaning with argon ions was carried out. The determination of the chemical composition was carried out using the ratios of the elements obtained in [15]. 

#### 2.3.2. Study of Porosity of Materials

The determination of the volumetric porosity of composite materials was carried out by the methods described below. The specific surface areas for composite materials based on HA and PLGA were estimated according to the data obtained on an automated 3Flex sorption (Micromeritics, Norcross, GA, USA). Before the experiment, all samples were degassed in vacuum (10^−2^ Top) at 80 °C for 2 h.

The total volumetric porosity of the materials was determined by the method of hydrostatic weighing. The dried samples were weighed and saturated with water (a liquid that wet the sample). Then the test sample was weighed in a saturating liquid, the pore volume was determined from the difference in the sample mass in liquid and in air [16].

The study of the diffusion properties of composites (characterization of the open porosity of materials) was carried out on a Franz diffusion cell with rhodamine B. The concentration of rhodamine B was determined on a UVmini1240 SHIMADZU spectrophotometer (Shimadzu, Kyoto, Japan). To construct the calibration line, we used the concentration of rhodamine B solutions from 0.013 to 13 mg/L [17]. 

#### 2.3.3. Study of the Surface Characteristics of Materials

To determine the concentration of acid–base centers on the surface of composite materials, the Hammett indicator method was used [18]. Weak organic acids and bases were used as acid–base indicators, which have different colors of molecular and ionic forms due to tautomeric rearrangements during the dissociation of the indicator with pKa ranging from −5 to 20. The concentration of indicators was determined spectrophotometrically using a PE 5400UF instrument (ECROSCHEM, St. Petersburg, Russia). The contact angle of wetting of composite materials in contact with water and glycerol was measured on an EasyDrop, Kruss device by the lying drop method.

### 2.4. In Vitro Methods

Assessment of the viability of the immune system cells after incubation on the surface of the test materials was performed by the following procedure: during the analysis, monocytes isolated from human blood were first inoculated onto the samples. Monocytes were isolated from the blood of three donors. Cell pellet was resuspended in Macrophage serum-free medium or X-Vivo at a concentration of 1 × 10^6^ cells per mL. Cells were seeded in 12-well plates with samples (2 mL per well) and stimulated with the cytokines INF-γ and IL-4 controlling the differentiation of macrophages (INF-γ (PEPROTECH): 100 ng/mL, IL-4 (PEPROTECH): 10 ng/mL). Then, the samples were incubated at 37 °C for 6 days. After that, the supernatant was taken from each well, leaving 500 µL of medium with cells in the well. AlamarBlue reagent (50 µL AlamarBlue/cell medium volume ratio 1/10) was added to the wells. Cells with AlamarBlue were incubated for 3 h at 37 °C in a dark place. After incubation, the cell medium with AlamarBlue was added to a 96-well plate (three wells for each sample). The intensity of the fluorescence signal was measured using a Tecan Infinite 200 microreader (Tecan Group Ltd., Mannedorf, Switzerland) at a wavelength of 540 nm.

## 3. Results and Discussion

### 3.1. Composition Porous HA Frameworks and Composites HA-PLGA

**Figure 1:**
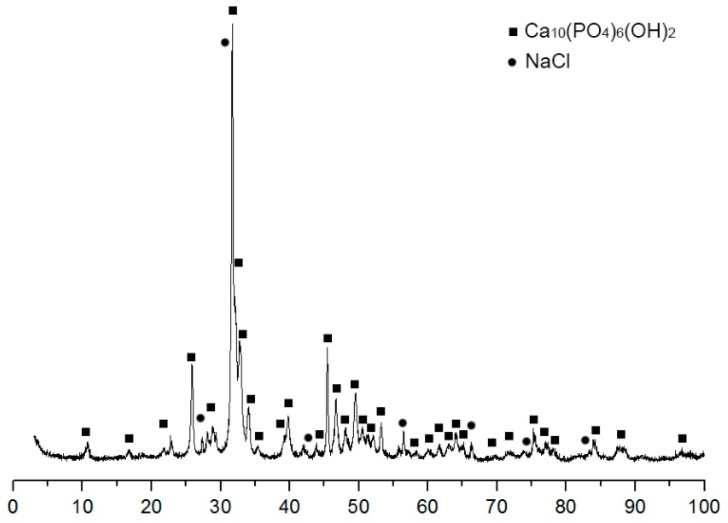
Diffraction pattern of the initial mixture of HA 75 wt.% and NaCl 25 wt.%.

When obtaining porous HA frameworks, each stage was carried out with control of the phase composition. The diffractogram of the initial mixture of HA + pore former (Figure 1) shows the presence of two phases in its composition—halite (NaCl) and hydroxyapatite (Ca_10_(PO_4_)_6_(OH)_2_), no foreign phases were found in the mixture.

The content of NaCl in the samples ranged from 10 to 50 wt.%. The HA-NaCl ratios are shown in Table 1.

**Figure 2:**
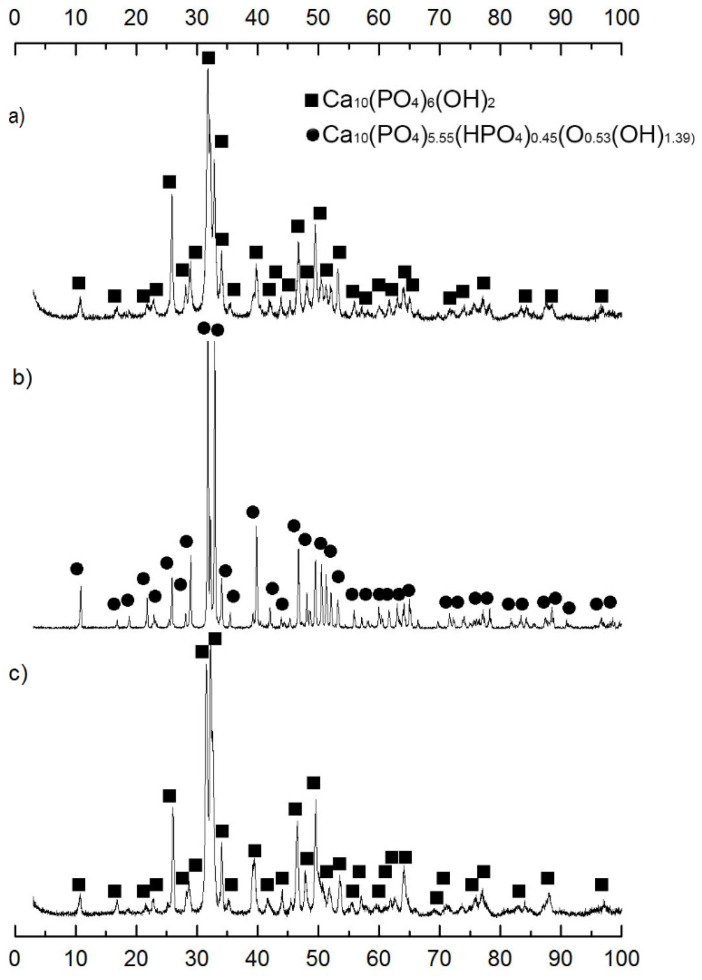
Diffraction patterns of porous frameworks (**a**) HA (90), (**b**) HA (75), (**c**) HA (50).

Controlling the phase composition of the resulting frameworks showed that the phase composition of the HA material (75) changed after calcination and removal of NaCl; the main phase was hydroxyapatite with the composition Ca_10_(PO_4_)_5,55_(HPO_4_)_0,45_(O_0,53_(OH)_1,39_) (Figure 2). In the composition of HA (90) and HA (50), the phase of hydroxyapatite was registered Ca_10_(PO_4_)_6_(OH)_2_. 

With an increase in the amount of NaCl, the lattice parameters and coherent scattering region (CSR) of the samples change (Table 2). Depending on the amount of NaCl, the phase ratio changes, which leads to a different final structure of the hydroxyapatite framework.

**Figure 3:**
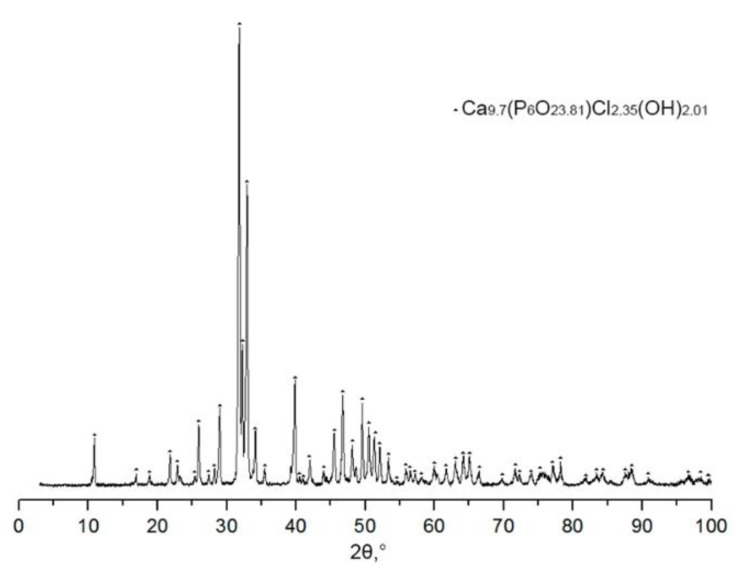
Diffractogram of HA (75) after calcination HA with NaCl.

We found that in the course of investigating the intermediate stage of preparation after calcining the framework in the process of partial incorporation of NaCl into the HA structure, a new phase of chlorine-substituted HA Ca_9.7_(P_6_O_23.81_)Cl_2.35_(OH)_2.01_ is formed (Figure 3), Ca_10_(PO_4_)_6_(OH)_2_ and NaCl were also observed in the composition.

After calcination, sodium chloride in the composition of the ceramic was subjected to dissolution and observed the formation of a new phase Ca_10_(PO_4_)_5.55_(HPO_4_)_0.45_(O_0.53_(OH)_1.39_). It can be assumed that the process of the formation of a new phase occurs according to the scheme:

$$C_{a10}{\left({\mathrm{PO}}_{4}\right)}_{6}{\left(\mathrm{OH}\right)}_{2}\underset{+NaCl}{\to }C_{a9.7}\left(P_{6}O_{23.81}\right){\mathrm{Cl}}_{2.35}{\left(\mathrm{OH}\right)}_{2.01}\underset{+H2O}{\to }C_{a10}{\left({\mathrm{PO}}_{4}\right)}_{5.55}{\left({\mathrm{HPO}}_{4}\right)}_{0.45}\left(O_{0.53}({\mathrm{OH}}_{1.39})\right)$$

Since the final phase contains hydrophosphate acid residues, we can assume that during the dissolution of NaCl, the hydrolytic decomposition of the compound Ca_9.7_(P_6_O_23.81_)Cl_2.35_(OH)_2.01_ occurs with the formation of a nonstoichiometric HA of the composition Ca_10_(PO_4_)_5.55_(HPO_4_)_0.45_(O_0.53_(OH)_1.39_), the crystallite size remains practically unchanged. 

**Figure 4:**
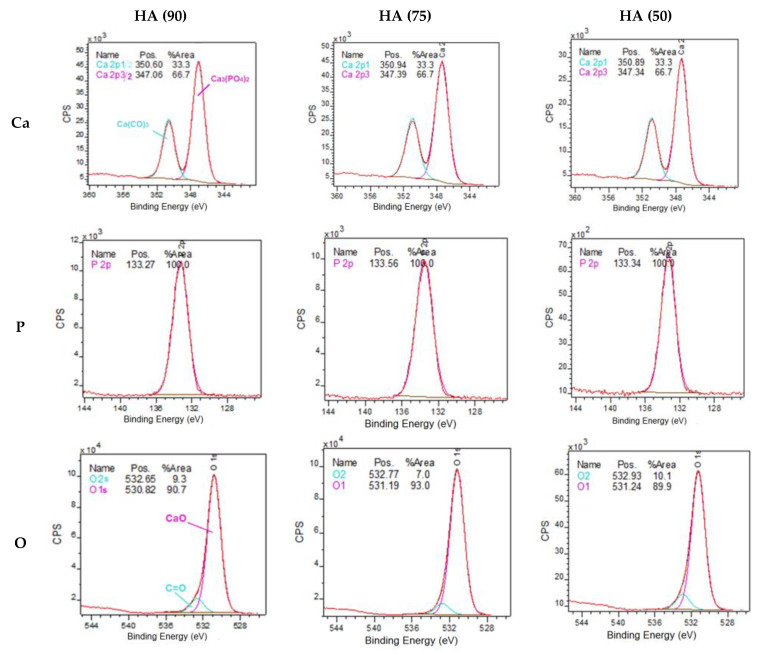
XPS spectra of Ca2p, P2p, and O1s of a porous ceramic surface ready for impregnation.

By the method of X-ray photoelectron spectroscopy, the chemical composition of the surface of samples of porous frameworks with different NaCl content was studied. Figure 4 shows the XPS spectra of Ca2p, P2p, and O1s of the porous ceramic surface ready for impregnation. The binding energies of Ca2p and P2p electrons and their ratios practically do not change, while the oxygen fraction and the ratio change in HA (75), which can be explained by its phase composition that is different from other materials. 

The difference in the elemental composition of the surface between HA (90)–HA (50) does not go beyond 1% (Table 3). The HA sample (75) has the closest to the literary Ca/P ratio for natural HA, which is also the closest to natural bone. The Ca/P ratios of real minerals differ significantly from the calculated one, since the calculation was performed minus the carbonates that are formed on the surface during the calcination of ceramics and are also adsorbed in the air atmosphere.

**Figure 5:**
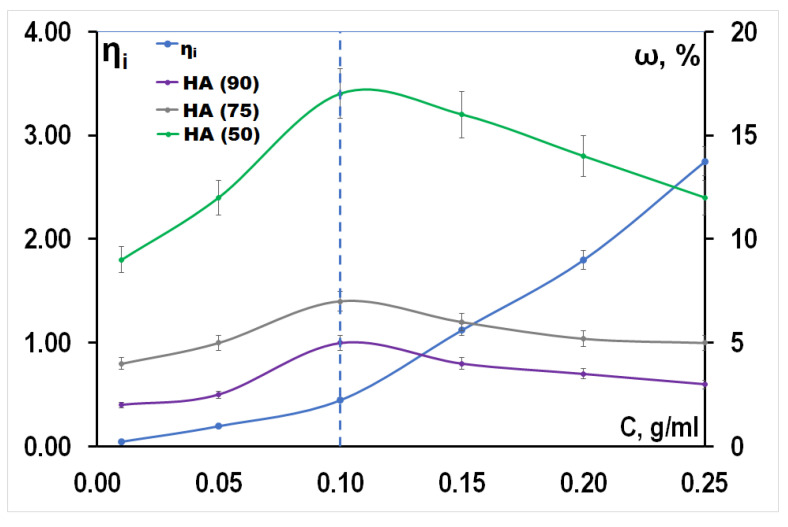
The dependence of the amount of PLGA, localized in the materials and the inherent viscosity by concentration of the solution.

To obtain composite materials, porous HA frameworks were impregnated with an PLGA solution. The concentration of PLGA was selected with the condition of determining the maximum weight gain of PLGA after impregnation (Figure 5). The concentration of PLGA affected the polymer content of the final composite (Figure 5). With an increase in the concentration of the solution, the proportion of PLGA in the materials increases, however, with an increase in the concentration above 0.1 g/mL, the proportion of PLGA in the materials decreases, which is associated with an increase in the viscosity of the solutions. The concentration at which occurs a change in slope of the curve inherent viscosity (η_i_) upward coincides with the point of the maximum mass fraction of PLGA in materials. The observed effect is explained by the fact that at concentrations above 0.1 g/mL the intermolecular interaction of the polymer molecules is amplified, high viscosity prevents the solution to penetrate more deeply into PLGA and secured in the frame HA. The porosity of materials also affects the amount of polymer in the material; with an increase in the amount of a pore former, an increase in the amount of PLGA in composites occurs. 

In summary, it should be noted that the amount of PLGA in the final composites increases from the HA (90) to the HA (50). This fact is associated with an increase in the porosity of the frameworks of the composites (Table 4). Ultrasonic treatment provides an increase in the proportion of PLGA in the material due to the sound capillary effect.

The use of ultrasound leads to a decrease in time costs by ≈72 times and an improvement in the quality of impregnation with a polymer by ≈40% relative to ordinary impregnation without ultrasound.

**Figure 6:**
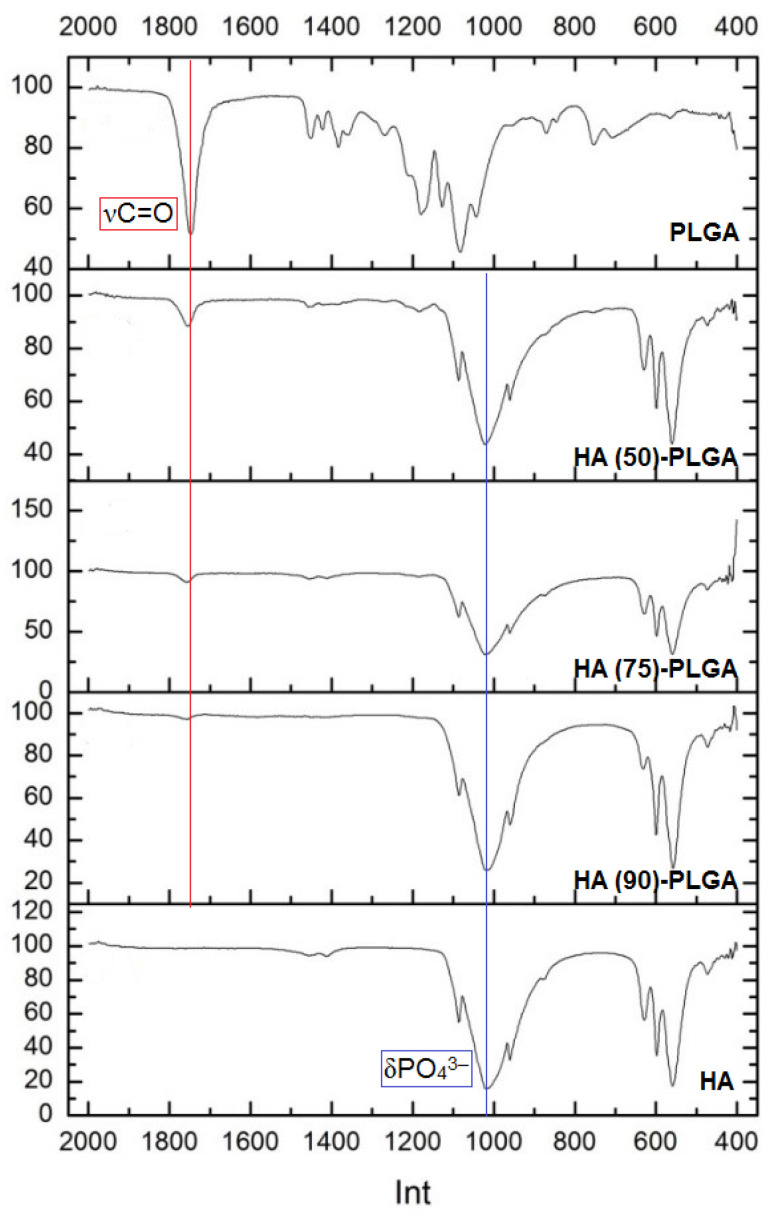
IR spectra of composites HA-PLGA and starting components.

The IR spectra (Figure 6) of the composites contain bands corresponding to the vibrations of the νC=O and δC–C bonds in the PLGA structure, as well as δPO_4_^3−^ included in the HA structure [20]. 

Due to the fact that the mass fraction of the copolymer is small, the intensity of the νCH and νCH_3_ bands is extremely low. In the spectra of composites, a slight shift of the carbonyl group band to shorter wavelengths occurs, which is usually associated with the geometry of the molecule, the mass of atoms associated with the carbonyl group, induction and mesomeric effects, steric factors [21], which suggests the formation of low-energy bonds between the PLGA and the surface HA.

### 3.2. Porosity of HA-PLGA Composites

**Figure 7:**
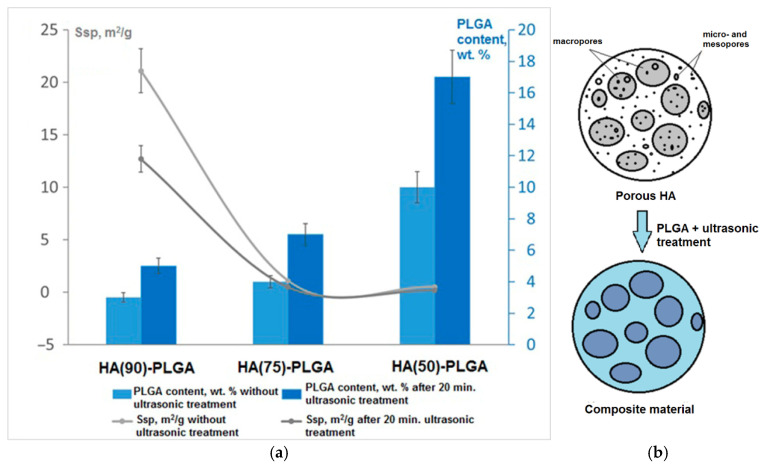
(**a**) The dependence of the specific surface area and the number of PLGA on the structure of the frameworks; (**b**) the scheme of coating the surface with materials.

The study of the specific surface area of the samples (Figure 7a) showed that after sonication there is a significant decrease in Ssp ≈ 50% relative to untreated samples, which is associated with the displacement of air that fills the ceramic frame. Probably, there is a partial overlap of micro- and mesopores (Figure 7b), while macroporosity is retained in the samples. After removing the material from the solution, the solvent evaporates and the surface of the ceramic is lined with a polymer to form a film that overlaps the microporosity but leaves the macroporosity (empty space capable of forming channels in the ceramic through which liquids penetrate into the material).

**Figure 8:**
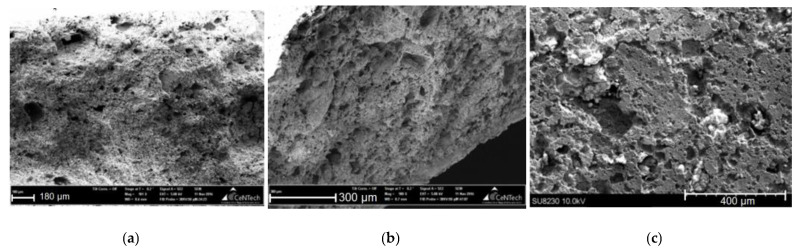
SEM images of fractures of composite materials (**a**) HA (90)-PLGA, (**b**) HA (75)-PLGA, (**c**) HA (50)-PLGA.

This statement is confirmed by scanning electron microscopy (SEM) images of sample fractures (Figure 8). The sections are covered with pores from 5 to 70 μm. There are also single pores up to 150 μm in size.

**Figure 9:**
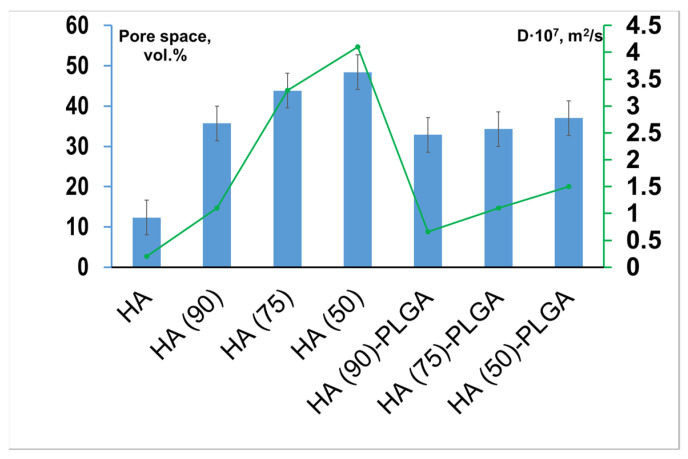
Open porosity of materials before and after impregnation with PLGA (vol.%) and values of the diffusion coefficient D (m^2^/s) for materials HA (90)-PLGA–HA (50)-PLGA and initial porous HA frameworks.

The apparent (open) porosity of the samples was determined by the liquid absorption method. Based on the results obtained, the open porosity was calculated for composites and initial non-impregnated frameworks (Figure 9). The volume of open porosity decreases after the ceramic is impregnated with PLGA due to the filling of a part of the pores. Moreover, the higher the initial porosity, the better the penetration of the polymer into the interior of the porous ceramic.

To confirm that the porosity of the samples is open, the diffusion coefficient was calculated using the Franz cell (Figure 9). Composites and frameworks were used as a membrane. This approach allows us to conclude that three structures were obtained in which open porosity is formed in different ratios. The data obtained are in direct relationship—with an increase in the porosity of materials, there is a regular increase in the diffusion coefficient D. 

### 3.3. Surface Properties of Composite Materials HA-PLGA

**Figure 10:**
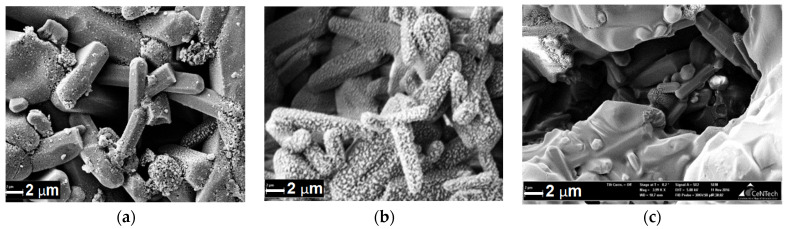
SEM images of the surface of composites (**a**) HA (90)-PLGA, (**b**) HA (75)-PLGA, (**c**) HA (50)-PLGA.

SEM images were obtained for the composites (Figure 10). All three composites are characterized by different surface polymer coating. HA (90)-PLGA has an uneven deficient coating with single polymer aggregates with a coverage volume of 10–50% of the surface in different areas. HA (75)-PLGA has a uniform island coverage of HA particles with a polymer over the entire surface area and the coverage volume is ≈68–70% in different areas, such a coating forms a large number of interphases. HA (50)-PLGA is completely covered by the PLGA, the coating is nonuniform, as evidenced by large PLGA aggregates on the ceramic surface.

**Figure 11:**
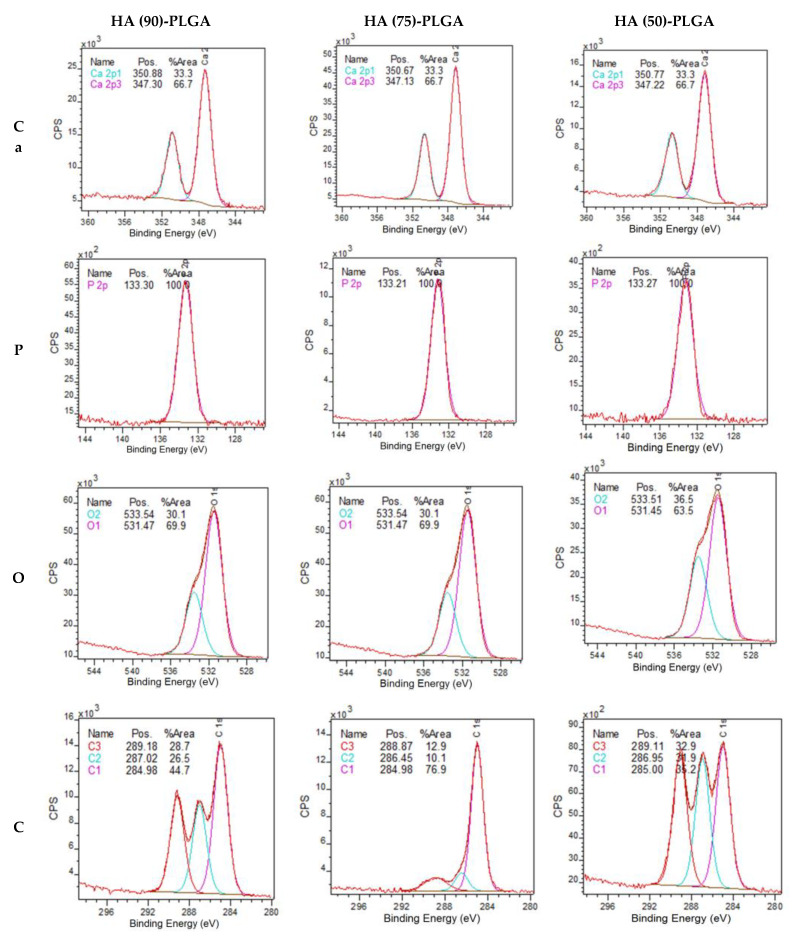
XPS spectra of Ca2p, P2p, O1s and C1s surfaces of composite materials.

The chemical composition of composite samples was studied by X-ray photoelectron spectroscopy (Figure 11). The binding energies of electrons Ca2p and P2p practically do not change when comparing the composites with each other, as in the case of pure ceramics, however, the O1s ratio changes in HA (75)-PLGA, which can be explained by its phase composition that differs from other materials.

The XPS results (Table 5) showed that on the surface of HA (75)-PLGA, the carbon content is significantly less than on the surface of HA (90)-PLGA and HA (50)-PLGA. This is probably due to the fact that the HA (75)-PLGA sample has a different phase composition from the others, which led to a change in the nature of the coating.

Comparing the composition of the surface after coating with a polymer with pure ceramics, it can be observed that the coating with a PLGA layer leads to a change in the Ca/P ratio on the surface; it significantly decreases with an increase in the amount of PLGA.

**Figure 12:**
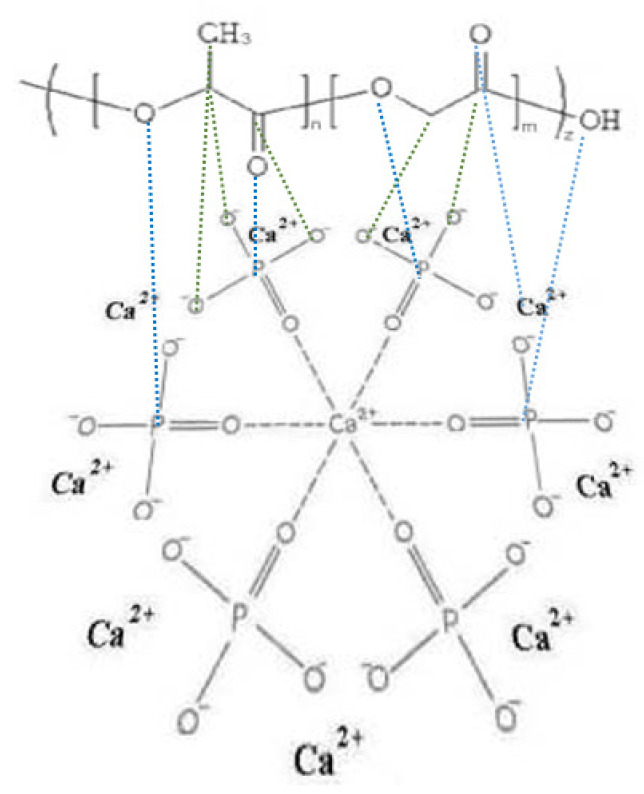
Scheme PLGA orientation of functional groups to the surface of HA.

The ratio of chemical bonds of carbon in composites changes (Table 6). In the HA (75)-PLGA samples, an increase in the area of the C1s peak corresponding to the C–C/C–H (1) bond and a decrease in the area of the C1s peak for the C–O/COOR (2) and (COOH) bond (3) are observed. It can be assumed that the decrease in the proportion of C–O/COOR (2) and (COOH) (3) bonds in the HA (75)-PLGA sample is due to the fact that the bonds are associated with HA at the interphases (according to SEM data, Figure 10). Since the Ca/P ratio in the initial HA ceramics (75) is somewhat lower than in the other samples, this suggests that a larger number of P atoms, which is more electronegative than Ca, allows a stronger association of PLGA molecules on the HA surface, as a result of which functional groups CO/COOR (2) and (COOH) (3) are oriented “inward” to HA (Figure 12). This fact leads to a significant decrease in the area of C2 and C3.

**Figure 13:**
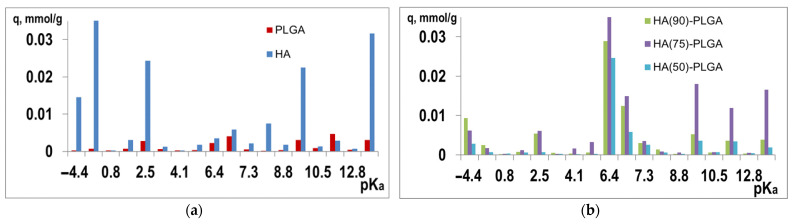
Distribution of ABC (q, mmol/g) (**a**) on the initial components and (**b**) on composites.

The analysis of functional centers on the surface of composite materials was carried out by the Hammett indicator method. The diagrams (Figure 13) show that pure HA has pronounced acid–base centers (ABC) with pK_a_ = −4.4; −0.29; 9.15; 16.8, represented by calcium and the oxygen lone pair of phosphate groups. The profile of the composites looks different from HA and PLGA. They also contain acid–base centers corresponding to HA and PLGA, but at the same time, intensely expressed new centers appear in the region of pK_a_ = 6.4–6.9, pK_a_ = 9.45, and pK_a_ = 12. The appearance of new centers is associated with weak interactions between the components of composite materials. Due to the orientation of PLGA relative to the HA centers in composite materials, their acid–base properties are enhanced. In composites, the concentration of acidic and basic centers increases, and there is a correlation between the results and the Ca/P ratio on the surface. The presence of a large number of interphases of composite materials promotes the formation of new centers, while the specific distribution of PLGA enhances this effect. 

For all composites and initial components, the contact angle (θ, °) was determined by the lying drop method. All materials and components are hydrophilic in nature, since θ_water_ < 90° (Table 7).

Calculation of the surface energy of the samples (Table 8) showed that HA (75)-PLGA also has the highest surface energy among composites, this is facilitated by the composition of its surface, on which the number of ABC in the region pK_a_ = 6.4 prevails, the centers are formed behind due to the greater amount of oxygen on the surface of the material, as well as due to the island coating of the ceramic with a polymer. 

**Figure 14:**
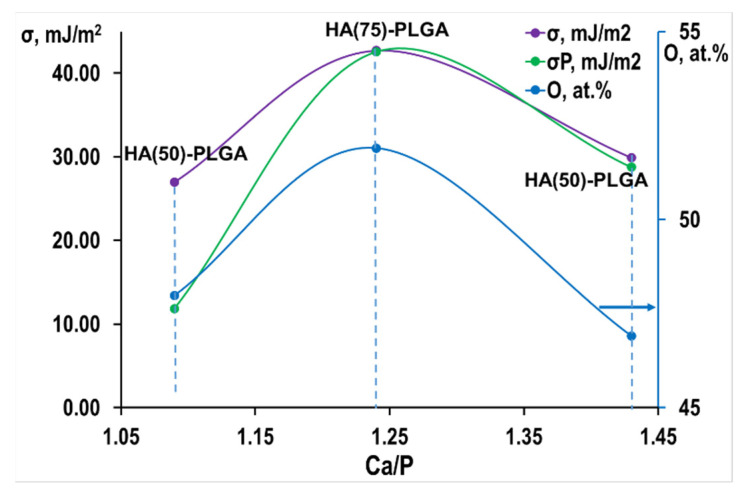
Oxygen content and the value of the polar component from the Ca/P ratio.

There is a codependency between σ^P^ and the oxygen concentration on the surface of materials—with an increase in the amount of oxygen, the polar component of the surface energy naturally increases (Figure 14).

The study of the acid–base properties of the surface showed that in the composites there is an increase in the concentration of acid–base centers, there is a codependency of the results with the Ca/P ratio. Calcium-deficient HA on the surface of materials promotes the formation of new centers, while the specific distribution of PLGA enhances this effect.

### 3.4. Viability, Pro- and Anti-Inflammatory Properties of Composite HA-PLGA Materials

Important functional properties such as biocompatibility and anti-inflammatory properties of HA-PLGA composites were studied by analyzing the developmental features of the cell-mediated immune response of individual donors in vitro by the nature of the production of pro- and anti-inflammatory cytokines by primary monocytic macrophages in the studied samples, depending on the direction of their differentiation in the process culturing CD14+ blood monocytes. 

**Figure 15:**
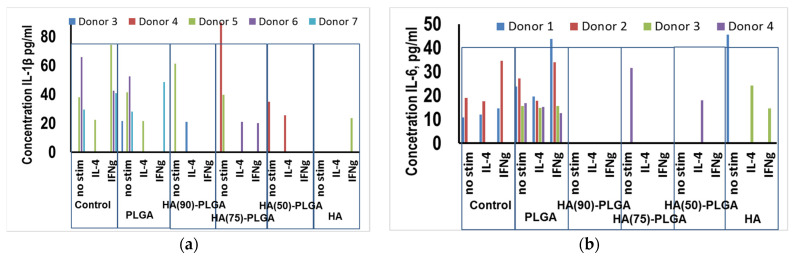
Influence of composite materials on the secretion of cytokines by primary human macrophages, depending on the direction of their differentiation (**a**) IL-1β; (**b**) IL-6.

The Enzyme-linked immunosorbent assay (ELISA) results showed that in the presence of PLGA, a significant increase in the secretion of IL-1β was recorded in the culture of M0-macrophages from donors 1 and 2 (Figure 15a). In the presence of HA, the production of IL-1β by M0-macrophages increases 4.5-fold relative to the control only in donor 1. M2 macrophages obtained by stimulating monocytes with IL-4 secrete IL-1β in large amounts on the sixth day of cultivation in the presence of PLGA in all donors and in the control in donors 1 and 2 (cells cultured on plastic). Upon stimulation of macrophages with IFNγ, the expression of IL-1β is similar, but it is more pronounced than in macrophages M2 (Figure 15a). As for the samples of HA (75)-PLGA and HA (50)-PLGA, no statistically significant parameters of IL-1β secretion were recorded in the presence of these composites, with the exception of donor 3, whose cells showed a proinflammatory cytokine response in the M0 culture in the presence of the sample. HA (75)-PLGA and culture M2, in the presence of HA (50)-PLGA, respectively, and in the control cells of this donor IL-1β did not secrete (Figure 15a).

The ELISA results showed that in the presence of the HA (75)-PLGA composite, an increase in the secretion of IL-6 in the culture of M0-macrophages (not activated macrophages) of donor 5 is observed, which may indicate the potential readiness of M0-macrophages for differentiation in the “proinflammatory” direction of M1 (Figure 15b). Increased production of IL-6 in comparison with control values was recorded in M0-macrophages of donor 5 in the presence of HA (90)-PLGA and PLGA. In donor 6, on the contrary, in the culture of M0-macrophages, a decrease in the production of IL-6 in the presence of PLGA was observed in comparison with the control values. In the presence of hydroxyapatite, IL-6 secretion in M0 macrophages was not detected in any of the donors, which indicates the absence of an acute inflammatory immune response (Figure 15b). During M2 activation of macrophages, statistically significant changes in IL-6 secretion were not found, regardless of the composition of the test sample (Figure 15b). With M1 activation, only donors 5 in the presence of HA and 6 in the presence of the sample HA (75)-PLGA show a significant decrease in the concentration of IL-6 in comparison with the control values. In donor 7, on the contrary, an increase in the production of IL-6 in M1 macrophages in the presence of PLGA was recorded both in comparison with the control and in comparison with its concentration in M0 macrophages.

Enzyme immunoassay showed that expression of CCL18 occurs in control in almost all donors with M2 activation of macrophages; insignificant secretion of this cytokine is also observed in control samples in M0 and M1 macrophages. Consequently, the cytokine-producing physiological function of M2 macrophages is preserved and is fully realized. In the presence of hydroxyapatite, a statistically significant level of CCL18 production was observed only in donor 7 with alternative M2 activation of macrophages. In the presence of composites and pure PLGA, expression of CCL18 was not observed regardless of the direction of differentiation of macrophages in any of the studied samples. This fact, on the one hand, indicates the absence of an active anti-inflammatory cytokine response during the interaction of innate immunity cells with samples of polymer materials. On the other hand, the cytokine network functions in such a way that the biological role of CCL18 in this case can be duplicated by other anti-inflammatory mediators. In addition, minimal cytokine production or its absence in a particular donor, regardless of the direction of differentiation of macrophages, may be due to the epigenetic mechanisms of the peculiarities of cytokine gene expression.

**Figure 16:**
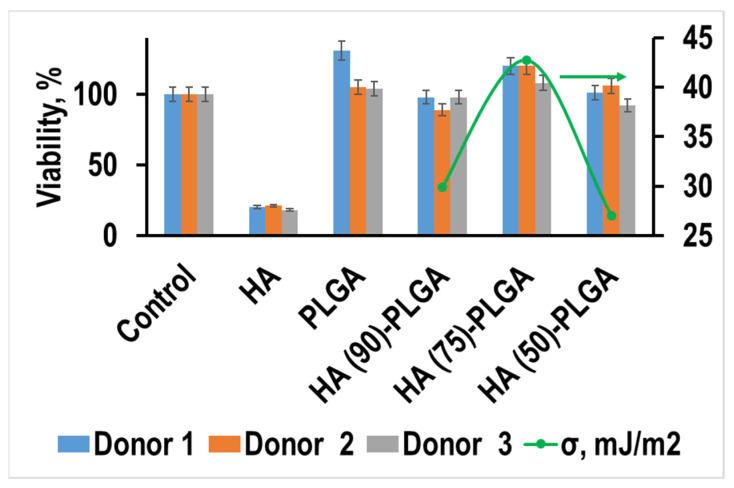
Viability of human macrophages on the 6th day of incubation in the presence of HA-PLGA composites and initial components.

The viability test showed (Figure 16) that the cells in the samples with PLGA and composites were generally comparable to the control sample. Cells in pure HA samples show low viability. Despite the fact that pure HA has pronounced cytotoxic properties, the addition of PLGA can significantly reduce the surface energy and improve the viability of cells.

The values of the viability correlate well with the value of the surface energy of materials and the concentration of acid–base centers in the pKa region of 6.4 and 6.9; this effect may indicate that it is these surface properties that are most important for these composites. These properties are determined by the phase composition of the initial HA ceramics and the nature of the PLGA distribution over its surface.

## 4. Conclusions

The conditions for obtaining porous hydroxyapatite frameworks with the participation of NaCl as a pore-forming agent were found (700 °C is the sintering temperature, the amount of NaCl is 10–50 wt.%). It is shown that the amount of pore former NaCl affects not only the porosity, but also the elemental composition of the surface and the ratio of phases in the samples. It was found that at a content of 25 wt.% NaCl, the main phase is nonstoichiometric hydroxyapatite with the composition Ca_10_(PO_4_)_5.55_(HPO_4_)_0.45_(O_0.53_(OH)_1.39_), (CSR 60 nm) with a ratio (Ca/P) = 1.49 on the surface characteristic of natural bone, and at other contents of the pore former NaCl, the main phase is stoichiometric Ca_10_(PO_4_)_6_(OH)_2_ with CSR parameters = 43 nm and (Ca/P) = 1.52 for a sample with 10 wt.% NaCl and CSR = 30 nm, (Ca/P) = 1.50 for material with 50 wt.% NaCl.

Composite materials were obtained in the ratio HA:PLGA 95:5 (HA (90)-PLGA), 93:7 (HA (75)-PLGA), 83:17 HA (50)-PLGA. It was found that at a PLGA concentration of 0, 1 g/mL and ultrasonic exposure, the maximum affinity between HA and PLGA is possible. The amount and localization of the polymer component, the elemental composition and surface energy of the composite samples are recovered by the structural characteristics of the HA frame: the size of the pore space, the phase and elemental composition of the surface. The mass of the applied PLGA is determined by the open porosity of the HA frame, and the maximum value is characteristic of the HA frame (50). With an increase in the amount of the polymer component, a decrease in the open porosity of the composite material relative to the initial HA-framework (35–48 vol.% to 32–37 vol.%) and a decrease in the diffusion coefficient (0.66 m^2^/s for HA (90)-PLGA and 1.5 m^2^/s for HA (50)-PLGA). 

Three types of localization of the polymer component in the HA-PLGA composite materials were established, depending on the ratio of the components and the porosity of the HA-composite: Type 1—uneven coating (10–50% of the surface) with the formation of island aggregates up to 7 µm, for HA (90)-PLGA; Type 2—uniform island coating (70% of the surface) with 50–200 nm of polymer particles for HA (75)-PLGA; Type 3—film coating (100% of the surface and coating thickness up to 2 µm) for HA (50)-PLGA and the value of surface energy for Type 2 of the coating of the composite material HA (90)-PLGA that has the highest value of 42.71 mJ/m^2^ was shown. For composite materials (especially of the second type), the concentration of acid–base centers increases significantly from pKa = 6.4, 6.9, 7.3, 12 to 0.088 mmol/L and decreases from pKa = −0.44, −0.29, 4.5, 9.45 and 16.8 to 0.0005 mmol/L, due to a change in the Ca/P/O ratio relative to the frameworks and the appearance of new polymer–ceramic interphases. In vitro studies have shown that despite the fact that pure HA has pronounced cytotoxic properties, the addition of PLGA can significantly reduce surface energy and improve the viability of materials.

## 5. Patents

Patent No. 2669554 Russian Federation, IPC A61L 27/10, A61L 27/58. A method of obtaining biodegradable composite materials with open porosity for bone tissue restoration D.N. Lytkina, A.A. Berezovskaya, I.A. Kurzina, N.M. Korotchenko, V.V. Kozik, applicant and patentee of GOU VPO “Tomsk State University”. Publ. 10.12.2018, Bul. No. 29. 5 p.

## Figures and Tables

**Table 1:** Composition of porous frameworks of materials based on hydroxyapatite and sodium chloride.

Sample	HA (90)	HA (75)	HA (50)
HA content in the initial mixture, wt.%	90	75	50
NaCl content in the initial mixture, wt.%	10	25	50

**Table 2:** Crystal parameters HA systems of different compositions.

Sample	Phase Composition	Crystal CellParameters, Å	CSR, nm
Initial HA	Ca_10_(PO_4_)_6_(OH)_2_, hexagonal P6_3_/m	a = 9.435279 b = 9.435279 c = 6.886613	50
HA (75) + NaCl after calcination	Ca_9,7_(P_6_O_23,81_)Cl_2,35_(OH)_2,01_, hexagonal P6_3_/m	a = 9.406083 b = 9.406083 c = 6.858610	59
HA (90)	Ca_10_(PO_4_)_6_(OH)_2_, hexagonal P6_3_/m	a = 9.518597 b = 9.518597 c = 6.843739	43
HA (75)	Ca_10_(PO_4_)_5,55_(HPO_4_)_0,45_(O_0,53_(OH)_1,39_), hexagonal P6_3_/m	a = 9.406839 b = 9.406839 c = 6.873786	60
HA (50)	Ca_10_(PO_4_)_6_(OH)_2_, hexagonal P6_3_/m	a = 9.419352 b = 9.419352 c = 6.880314	30

**Table 3:** Elemental composition of the surface of porous HA frameworks after dissolution of NaCl, obtained by XPS, depth 5–10 nm.

Sample	Element Content on the Surface, at. %			
	Ca	O	P	Ca/P
HA (90)	25.3	58	16.6	1.52
HA (75)	25.2	57.9	16.8	1.49
HA (50)	24.7	58.7	16.4	1.50
HA lib.	Ca/P calculated = 1.67			1.48 [19]
Bone	-			1.49

**Table 4:** The ratio of components in composite HA-PLGA.

Composite	PLGA Content, wt. % after 24 h Impregnation without Ultrasonic Treatment	PLGA Content, wt. % after 20 min. Ultrasonic Treatment
HA (90)-PLGA	3	5
HA (75)-PLGA	4	7
HA (50)-PLGA	10	17

**Table 5:** Elemental composition of the surface of composites, thickness 5–10 nm.

Sample	Element Content on the Surface, at. %				
	C	Ca	O	P	Ca/P
HA (90)-PLGA	31.77	12.58	46.90	8.75	1.43
HA (75)-PLGA	12.00	20.00	51.90	16.10	1.24
HA (50)-PLGA	18.35	17.57	47.98	16.10	1.09

**Table 6:** Carbon composition of the surface of composites, depth 5–10 nm.

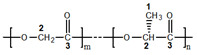	Fraction of C1s in the XPS Spectrum, at. %		
	C1 (≈285.0 eV) → Aliphatic Carbon (C–C/C–H)	C2 (≈286.8 eV) → Alcohol Group (C–O/COOR *)	C3 (≈288.9 eV) → Carboxyl Group (C * OOR) (≈289.3 eV) → Carboxylic Acid (COOH), O (C–O) O, (C=O) O (C=O)
HA (90)-PLGA	49	40	11
HA (75)-PLGA	83	8	9
HA (50)-PLGA	44	25	31

* аliphatic radical.

**Table 7:** Values of the contact angle θ, ° for the initial components and composites of HA-PLGA with water, glycerol.

Sample	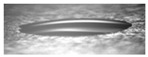 HA	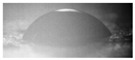 PLGA	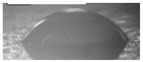 HA (90)-PLGA	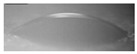 HA (75)-PLGA	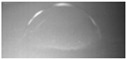 HA (50)-PLGA
θwater,°	14.1 ± 0.80	65.2 ± 1.92	54.0 ± 0.61	33.0 ± 2.31	85.0 ± 1.03
θglycerol,°	17.6 ± 6.18	49.1 ± 5.32	72.3 ± 2.54	90.8 ± 4.69	85.7 ± 3.81

**Table 8:** Dispersion (σ^D^) and polar (σ^P^) components of the surface energy of materials.

Sample	σ^D^, mJ/m^2^	σ^P^, mJ/m^2^	σ, mJ/m^2^
HA	12.25 ± 8.80	62.50 ± 23.22	74.75 ± 32.02
PLGA	21.47 ± 1.99	22.33 ± 1.43	43.80 ± 3.42
HA (90)-PLGA	1.16 ± 1.16	28.75 ± 1.47	29.91± 1.83
HA (75)-PLGA	0.14 ± 0.16	42.57 ± 2.33	42.71 ± 2.49
HA (50)-PLGA	15.17 ± 0.99	11.83 ± 0.78	27.00 ± 1.77

## Data Availability

The datasets used and/or analyzed during the current study are available from the corresponding author on reasonable request.
